# Supplementary material for: The role of playgrounds in the development of children’s fundamental movement skills: A scoping review
Source: PLoS One. 2023 Dec 13;18(12):e0294296. doi: 10.1371/journal.pone.0294296 (PMC10718446; doi:10.1371/journal.pone.0294296)
Supplement: Attachment — Submitted filename: PONE-D-23-14269_Reviewer Feedback.pdf [file pone.0294296.s003.pdf]

PLOS ONE

The role of playgrounds in the development of children's fundamental movement skills: A scoping review

--Manuscript Draft--

**Manuscript Number:**	PONE-D-23-14269
**Article Type:**	Research Article
**Full Title:**	The role of playgrounds in the development of children's fundamental movement skills: A scoping review
**Short Title:**	Scoping review of playgrounds and fundamental movement skills
**Corresponding Author:**	Charlotte Skau Pawlowski<br>University of Southern Denmark<br>Odense M, DENMARK
**Keywords:**	fundamental movement skills; motor development; children; playground; Physical activity
**Abstract:**	Fundamental movement skills are the basic skills children should develop but are low in children from high-income countries. Literature indicates that playgrounds can play an important role challenging children's balance, agility, and coordination. However, knowledge on the influence of playgrounds on children's fundamental movement skills development is fragmented. The aim of the present scoping review was to create an overview of all research that is relevant when studying the influence of unstructured playground play on children's fundamental movement skills. Four electronic databases (Scopus, Web of Science, SportDiscus, and PsycInfo) were searched systematically in May 2022 following the PRISMA guidelines, leading to a final set of twelve publications meeting the inclusion criteria. The results of these publications indicate that it is important to design playgrounds with various features targeting balance, climbing, throwing, and catching to provide opportunities for children to enhance each fundamental movement skills (i.e., stability, locomotor skills, and object control skills). Also, spreading features over a large area of the playground seems to ensure ample space per child, stimulate children to use locomotor skills by moving to and from features, and to play active games without equipment. Possibly, also natural play settings develop children's fundamental movement skills. These findings, however, should be read with caution. More experimental studies using objective and standardized fundamental movement skills tests are needed in this research field for a more robust conclusion.
**Order of Authors:**	Charlotte Skau Pawlowski
	Cathrine Damsbo Madsen
	Mette Toftager
	Thea Toft Amholt
	Jasper Schipperijn
**Additional Information:**	
**Question**	**Response**
**Financial Disclosure**<br><br>Enter a financial disclosure statement that describes the sources of funding for the work included in this submission. Review the submission guidelines for detailed requirements. View published research articles from *PLOS ONE* for specific examples.	

Powered by Editorial Manager® and ProduXion Manager® from Aries Systems Corporation

This statement is required for submission and **will appear in the published article** if the submission is accepted. Please make sure it is accurate.<br><br>**Unfunded studies**<br>Enter: *The author(s) received no specific funding for this work.*<br><br>**Funded studies**<br>Enter a statement with the following details:<br>• Initials of the authors who received each award<br>• Grant numbers awarded to each author<br>• The full name of each funder<br>• URL of each funder website<br>• Did the sponsors or funders play any role in the study design, data collection and analysis, decision to publish, or preparation of the manuscript?<br>• **NO** - Include this sentence at the end of your statement: *The funders had no role in study design, data collection and analysis, decision to publish, or preparation of the manuscript.*<br>• **YES** - Specify the role(s) played.<br><br>* typeset	
**Competing Interests**<br><br>Use the instructions below to enter a competing interest statement for this submission. On behalf of all authors, disclose any competing interests that could be perceived to bias this work—acknowledging all financial support and any other relevant financial or non-financial competing interests.<br><br>This statement is required for submission and **will appear in the published article** if the submission is accepted. Please make sure it is accurate and that any funding sources listed in your Funding Information later in the submission form are also declared in your Financial Disclosure statement.<br><br>View published research articles from *PLOS ONE* for specific examples.	

NO authors have competing interests

Enter: *The authors have declared that no competing interests exist.*

Authors with competing interests

Enter competing interest details beginning with this statement:

I have read the journal's policy and the authors of this manuscript have the following competing interests: [insert competing interests here]

* typeset

Ethics Statement

Enter an ethics statement for this submission. This statement is required if the study involved:

- Human participants
- Human specimens or tissue
- Vertebrate animals or cephalopods
- Vertebrate embryos or tissues
- Field research

Write "N/A" if the submission does not require an ethics statement.

General guidance is provided below. Consult the submission guidelines for detailed instructions. **Make sure that all information entered here is included in the Methods section of the manuscript.**

Powered by Editorial Manager® and ProduXion Manager® from Aries Systems Corporation

**Format for specific study types**<br><br>**Human Subject Research (involving human participants and/or tissue)**<br>• Give the name of the institutional review board or ethics committee that approved the study<br>• Include the approval number and/or a statement indicating approval of this research<br>• Indicate the form of consent obtained (written/oral) or the reason that consent was not obtained (e.g. the data were analyzed anonymously)<br><br>**Animal Research (involving vertebrate animals, embryos or tissues)**<br>• Provide the name of the Institutional Animal Care and Use Committee (IACUC) or other relevant ethics board that reviewed the study protocol, and indicate whether they approved this research or granted a formal waiver of ethical approval<br>• Include an approval number if one was obtained<br>• If the study involved *non-human primates*, add *additional details* about animal welfare and steps taken to ameliorate suffering<br>• If anesthesia, euthanasia, or any kind of animal sacrifice is part of the study, include briefly which substances and/or methods were applied<br><br>**Field Research**<br><br>Include the following details if this study involves the collection of plant, animal, or other materials from a natural setting:<br>• Field permit number<br>• Name of the institution or relevant body that granted permission	
**Data Availability**<br><br>Authors are required to make all data underlying the findings described fully available, without restriction, and from the time of publication. PLOS allows rare exceptions to address legal and ethical concerns. See the PLOS Data Policy and FAQ for detailed information.	Yes - all data are fully available without restriction

A Data Availability Statement describing where the data can be found is required at submission. Your answers to this question constitute the Data Availability Statement and **will be published in the article**, if accepted.<br><br>**Important:** Stating 'data available on request from the author' is not sufficient. If your data are only available upon request, select 'No' for the first question and explain your exceptional situation in the text box.<br><br>Do the authors confirm that all data underlying the findings described in their manuscript are fully available without restriction?	
**Describe where the data may be found in full sentences. If you are copying our sample text, replace any instances of XXX with the appropriate details.**<br><br>• If the data are **held or will be held in a public repository**, include URLs, accession numbers or DOIs. If this information will only be available after acceptance, indicate this by ticking the box below. For example: *All XXX files are available from the XXX database (accession number(s) XXX, XXX.*).<br>• If the data are all contained **within the manuscript and/or Supporting Information files**, enter the following: *All relevant data are within the manuscript and its Supporting Information files.*<br>• If neither of these applies but you are able to provide **details of access elsewhere**, with or without limitations, please do so. For example:<br><br>*Data cannot be shared publicly because of [XXX]. Data are available from the XXX Institutional Data Access / Ethics Committee (contact via XXX) for researchers who meet the criteria for access to confidential data.*<br><br>*The data underlying the results presented in the study are available from (include the name of the third party*	Data in the review is 12 publications owned by a third party and thus authors do not have permission to share the data

and contact information or URL).<br>• This text is appropriate if the data are owned by a third party and authors do not have permission to share the data.<br><br>* typeset	
Additional data availability information:	Tick here if your circumstances are not covered by the questions above and you need the journal’s help to make your data available.

Powered by Editorial Manager® and ProduXion Manager® from Aries Systems Corporation

Manuscript
Click here to access/download;Manuscript;The role of playgrounds in the development of children.docx

The role of playgrounds in the development of children's fundamental movement skills: A scoping review

Charlotte Skau Pawlowski[1,2*], Cathrine Damsbo Madsen[1,2], Mette Toftager[1,2], Thea Toft Amholt[3], Jasper Schipperijn[1,2]

[1]Research Unit for Active Living, Department of Sports Science and Clinical Biomechanics, University of Southern Denmark, Odense, Denmark

[2]Playground Research Center, University of Southern Denmark, Odense Denmark

[3]Center for Clinical Research and Prevention, Frederiksberg Hospital, Frederiksberg, Denmark

* Corresponding author: Charlotte Skau Pawlowski, cspawlowski@health.sdu.dk

Abstract

Fundamental movement skills (FMS) are the basic skills children should develop but are low in children from high-income countries. Literature indicates that playgrounds can play an important role challenging children's balance, agility, and coordination. However, knowledge on the influence of playgrounds on children's FMS development is fragmented. The aim of the present scoping review was to create an overview of all research that is relevant when studying the influence of unstructured playground play on children's FMS. Four electronic databases (Scopus, Web of Science, SportDiscus, and PsycInfo) were searched systematically in May 2022 following the PRISMA guidelines, leading to a final set of twelve publications meeting the inclusion criteria. The results of these publications indicate that it is important to design playgrounds with various features targeting balance, climbing, throwing, and catching to provide opportunities for children to enhance each FMS (i.e., stability, locomotor skills, and object control skills). Also, spreading features over a large area of the playground seems to ensure ample space per child, stimulate children to use locomotor skills by moving to and from features, and to play active games without equipment. Possibly, also natural play settings develop children's FMS. These findings, however, should be read with caution. More experimental studies using objective and standardized FMS tests are needed in this research field for a more robust conclusion.

Introduction

Fundamental movement skills (FMS) are the basic skills children should be competent at, such as stability skills (e.g., sitting, standing, balancing), locomotor skills (e.g., running, jumping, climbing), and object control skills (e.g., throwing, catching) [1]. Preschool years are crucial in terms of developing various FMS [2]. Developing proficiency in these skills is important, as FMS provide an underlying base for successful participation in physical activities across the life course [1, 3, 4]. Despite the importance of FMS for physical activity, FMS are generally low in preschool and school aged children from high-income countries [5]. Also, a recent systematic review showed that children (3-10 years old) in 25 countries were not achieving the FMS competence required to successfully participate in physical activity [6]. The ability to perform and master different types of FMS has also been associated with school readiness and performance [7, 8], social interaction with peers [9], and self-perception [10].

To increase FMS, many interventions have been developed in various contexts [11-13], but few FMS interventions have been implemented at scale [14]. Adult-directed, structured FMS programs are considered effective in developing children's FMS [15]. However, structured programs require educated staff and thus are expensive to conduct. Further, specific training only affects the development of the specific task trained and not necessarily other tasks related to the same FMS competence [16].

Literature also indicates that unstructured play at playgrounds is important for children's physical development, challenging their movement abilities such as balance, agility, coordination, and spatial awareness [17, 18]. This is also supported by the World Health Organization stating that active play and opportunities for unstructured physical activity can contribute to the development of motor skills in children under five years [19]. However,

playgrounds can be designed very differently, vary in size, features, and be built in conventional or natural materials. Further, studies addressing the influence of playgrounds on children's FMS development are carried out in different research fields with varying study designs, methods, and outcomes. A review of active play interventions aimed at promoting physical activity and FMS conducted in 2016 only included four studies [20], and could not draw firm conclusion due to the small number of eligible studies and their heterogeneity. Thus, knowledge on the role of playgrounds in children's FMS development is fragmented and a coherent overview of relevant research in this field is needed to help guide future FMS interventions [21, 22].

The aim of the present article was to create a global, interdisciplinary overview of relevant research investigating the influence of unstructured playground play on children's FMS to be able to identify and outline existing knowledge in this research field and hereby identify knowledge gaps and set the agenda for future research. To do this, we will conduct a comprehensive scoping review.

Materials and methods

The scoping review was conducted in accordance with the JBI methodology for scoping reviews [23] and the PRISMA guidelines were followed [24]. The review protocol was registered at Open Science Framework (https://doi.org/10.17605/OSF.IO/UYN2V) in May 2022.

The current scoping review is part of a broader project synthesizing evidence on the relationship between unstructured playground play and physical, mental, and social health among children and adolescents. Findings for other health outcomes than FMS when using playgrounds will be presented in separate publications.

Search strategy

Four electronic databases, Scopus, Web of Science, SportDiscus, and PsycInfo, were searched systematically in May 2022. Search terms were tested and revised by all authors (having expertise in the research field) in collaboration with a research librarian from the University of Southern Denmark. It was decided to create a comprehensive search strategy with two search blocks to obtain all relevant research on the topic. One search block contained all identified synonyms for ‘playground’, the other contained all identified synonyms for ‘children’. In Table 1, the search terms are shown for Scopus. The search terms were slightly adapted to fit each database.

Table 1. Search terms for Scopus

Table 2 Search terms for Scopus

(TITLE (playground*)) OR (((TITLE-ABS (schoolyard* OR "school ground*") OR AUTHKEY (schoolyard* OR "school ground*")) OR (TITLE-ABS (play W/3 (area* OR space* OR environment* OR field* OR natural OR nature OR outdoor OR place* OR structure* OR equipment OR park*)) OR AUTHKEY (play W/3 (area* OR space* OR environment* OR field* OR natural OR nature OR outdoor OR place* OR structure* OR equipment OR park*))) OR (TITLE-ABS ((school* OR daycare* OR "day care" OR childcare OR "child care" OR kindergarten*) W/6 (play OR playable OR played OR playing OR "physical* activ*" OR "organi?ed activit*" OR "unorgani?ed activit*" OR "structured activit*" OR "unstructured activit*" OR "recreation* activit*" OR "leisure activit*" OR "outdoor activit*" OR "vigorous activit*")) OR AUTHKEY ((school* OR daycare* OR "day care" OR childcare OR "child care" OR kindergarten*) W/6 (play OR playable OR played OR playing OR "physical* activ*" OR "organi?ed activit*" OR "unorgani?ed activit*" OR "structured activit*" OR "unstructured activit*" OR "recreation* activit*" OR "leisure activit*" OR "outdoor activit*" OR "vigorous activit*"))) OR (TITLE-ABS (playfield* OR playplace* OR playscape* OR playspace* OR "public open space") OR AUTHKEY (playfield* OR playplace* OR playscape* OR playspace* OR "public open space")) OR (INDEXTERMS (playground)) OR (ABS (playground*) OR AUTHKEY (playground*))) AND ((TITLE-ABS (adolescen* OR baby OR boy OR schoolboy* OR boyhood OR girlhood OR child* OR schoolchild* OR girl OR schoolgirl* OR infan* OR juvenil* OR kid OR minor OR newborn* OR new-born* OR paediatric* OR pediatric* OR preschool* OR puber* OR pubescen* OR teen* OR tween* OR toddler* OR youth* OR student* OR schoolage*) OR AUTHKEY (adolescen* OR baby OR boy OR schoolboy* OR boyhood OR girlhood OR child* OR schoolchild* OR girl OR schoolgirl* OR infan* OR juvenil* OR kid OR minor OR newborn* OR new-born* OR paediatric* OR pediatric* OR preschool* OR puber* OR pubescen* OR teen* OR tween* OR toddler* OR youth* OR student* OR schoolage*)) OR (INDEXTERMS (child) OR INDEXTERMS (adolescent) OR INDEXTERMS (pediatric))))

Selection criteria

For publications to be included in the current scoping review, the study needed to take place on a playground. Playgrounds were defined as a place designed or designated to facilitate play. For this review, we included publicly available outdoor playgrounds e.g., in parks or

neighborhoods, as well as playgrounds at early childhood education and care (ECEC) centers, schools, and healthcare centers. Publications that only examined sports facilities such as soccer fields, parkour parks, basketball courts, beach volley, etc. were excluded. Further, we differentiated between fixed (e.g., swings, monkey bars, trees) and portable playground features (e.g., balls and ropes). Publications only focusing on portable features were excluded. We included publications examining children aged 0-17. Also, we included all publications regardless of the children's health condition and physical abilities. We included scientific publications using all forms of study designs. Book reviews, conference abstracts, protocol papers, PhD dissertations and method development publications were excluded. Studies of unstructured play at the playground were included whereas studies only focusing on peer- and adult-led activities such as physical education, organized activities, or supervision on the playground were excluded as well as studies about playground policy. Articles that were peer-reviewed, published from January 2000 to May 2022, and written in English were included. There was no limit on country or origin of publications. Further, in the present review, studies were only included if FMS was one of the outcomes measured.

Selection procedure

All references were imported to Endnote 20.0.1 where duplicates were removed by one researcher (CM) and uploaded to Covidence. Then all authors screened the publications' title and abstract (Jun-Aug 2022) whereafter each full-text was assessed by two of the authors independently (Aug-Oct 2022). Conflicts were solved by one of two authors (CSP or JS). The reference list of 10 randomly chosen included publications were screened by one author (CM) for additional publications of relevance (Jan 2023). Due to the comprehensive search strategy, this did not result in additional publications being included. Therefore, we did not check all reference lists for included publications.

For the present review, all full-text publications investigating the association between unstructured playground play and FMS were selected. Relevant citations in these publications were screened but no additional publications of relevance were found. The results of the search and the study inclusion process are presented in a PRISMA flowchart, Fig 1.

Fig 1. Flowchart for selected publications

[See separate document]

Data extraction

For the current review, data extraction of the selected publications with FMS as a health outcome was completed by the first author (CSP) and cross-checked by MT. Data extracted included the aim of the publication, study design, participants, setting, health outcomes, methods used to measure FMS, and key findings relevant to the review, as well as general information about the study such as author, country, and year of publication.

Results

As seen in the PRISMA flowchart (Fig 1), the total number of hits was 66,279 related to the broad search on physical, social, and mental health outcomes in relation to children's playground use. After duplicates were removed 42,110 publications remained, of which 39,721 irrelevant titles and abstracts were excluded leaving 2,389 full-text publications to be screened. 1,941 full-text publications were excluded with reasons detailed in Fig 1. A total of 222 publications met the inclusion criteria, of which, 12 included FMS as at least one of the health outcomes investigated. Extracted data from the 12 included publications can be found in Table 2.

Table 2. Data extraction of the included publications

[See at back in the document because of length]

Characteristics of the studies

Three publications were from the USA [25-27]. Two publications each were from Australia [28, 29] and Norway [30, 31]. The remaining publications were from Italy [32], England [21], Canada [33], Indonesia [34], and Spain [35] (one study per country).

Two studies were published in the years 2000-2009 [30, 33], six studies were published between 2010-2019 [26, 27, 29, 32, 34, 35], and the remaining four studies were published from 2020 onwards (May 2022) [21, 25, 28, 31].

Half of the publications were conducted in early childhood education and care (ECEC) centers (n=6) [21, 25, 26, 30, 31, 34]. Three publications were conducted in a public area (i.e., neighborhood, park, sports center) [29, 32, 35]. Two publications were conducted at rehabilitation centers for children [27, 33], and one publication in primary schools [28].

Ten studies included children as participants (n=10), except two studies that included adults. One study included parents [35] and one study included parents and ECEC staff members [31]. These two studies focused on adults' perceptions on children's' FMS. Most of the child studies included a population of traditionally developed children (n=8) [21, 25, 26, 28-30, 32, 34]. One study included disabled children with Down syndrome [33] and one study included both typically developed and disabled children [27]. The age of the children studied varied widely across the publications included. Five of the studies included children from the age of three; 3-5 years (n=3) [21, 25, 26], 3-6 years (n=1) [34], and 3-15 years (n=1) [27]. Four studies included children from the age of five; 5 years (n=1) [32], 5-7 years (n=1) [30], 5-10 years (n=1) [29], and 5-12 years (n=1) [28]. One study investigated children aged 6-7 [33]. Socioeconomic status (SES) was infrequently reported and none of the studies had a focus on low SES population.

Ten study designs were cross-sectional without control groups [21, 25-29, 31, 33-35]. Two studies were intervention studies, both with an experimental group and a control group [30, 32]. All studies used quantitative methods. However, one study was a mixed methods study using qualitative adult interviews in combination with surveys [31].

Four of the publications solely measured FMS as an outcome [21, 25, 32], whereas the remaining eight publications investigated other health outcomes besides FMS such as social skills (n=3) [27, 31, 33], physical activity (n=3) [26, 29, 34], physical activity and physical fitness (n=1) [28], and weight status, social, and creative skills (n=1) [35].

Seven of the studies used test protocols to measure FMS, of which three studies used the Champs Motor Skills Protocol (CMSP) [21, 25, 26], one study used Pictorial Scale of Perceived Movement Skill Competence (PMCS) [34], one study used the Motor Fitness Test of the European Test of Physical Fitness (EUROFIT) [30], and two studies used selected items of validated FMS tests such as sprint run, vertical jump, side gallop, one leg balance, heel-to-toe walking, catch, and putting a medicine ball [28, 32]. Also, two studies each coded camera recordings [27, 33], and used survey [31, 35]. Loftesnes combined survey and interview. One study used the systematic observation method SOFIT [29].

Size of playground and fundamental movement skills

Two studies investigated the association between FMS and playground size in ECEC centers and primary schools, respectively. The cross-sectional study by True et al. 2017 including 229 children aged 3-5 from 22 ECEC centers in USA found a small positive relationship (effect size 0.33) between children's overall FMS competence and ECEC center playground size measured by Champs Motor Skills Protocol. Playground size was significantly associated with total motor score but not locomotor score and object control score individually [26]. In contrast, Grunseit et al. (2020)

found no association between playground size and FMS in a cross-sectional study FMS testing 210 children aged 5-12 in 43 Australian primary schools when adjusting for relevant covariates.

Playground setting and fundamental movement skills

Three studies investigated FMS in relation to the setting of the playground. In a cross-sectional study conducted in two urban and two rural ECEC center playgrounds in Indonesia including in total 66 3-6-year-old children, Famelia et al. [34] found no main effect of rural versus urban ECEC center playgrounds for locomotor skills and perceived movement skill competence even though children at the rural ECEC center playgrounds were found to be more sedentary than children in the urban ECEC center playgrounds. The two Norwegian studies also investigated the association between play in rural areas and FMS in a ECEC setting. The one study was a post-intervention study evaluating newly built nature playgrounds in nine ECEC centers. After being used for minimum two days a week in a one-year period, 12 parents being interviewed experienced their 2-6-year-old children being more able to cope with motor skills [31]. Fjørtoft [30] conducted an intervention study investigating 5-7-year-old children’s FMS after playing in a forest for 1-2 hours per day (experimental group of 46 children from one ECEC center) versus playing in a traditional ECEC center playground for 1-2 hours per day (control group of 29 children from two different ECEC centers). At the posttest 9 months after the pretest, significant differences were found in eight out of nine FMS test items in the experimental group (flamingo balance ($p<0.001$), plate tapping ($p<0.001$), standing broad jump ($p<0.001$), bent arm hang ($p<0.001$), Indian skip ($p<0.001$), sit-ups ($p<0.01$), beam walking ($p<0.01$), and shuttle run ($p<0.01$)) whereas the control group experienced a significant difference in three test items (standing broad jump ($p<0.01$), bent arm hang ($p<0.001$), and Indian skip ($p<0.001$). Thus, the motor fitness test showed a general tendency that the children using the forest as a playscape performed better in a variety of motor skills than the children on the traditional playground [30].

Playground features and fundamental movement skills

Eight studies investigated FMS in relation to playground features. Six of the studies included traditionally developed children in ECEC centers (n=3) and public playgrounds (n=3), whereas two studies focused on disabled children on a playground in a child rehabilitation center. The one ECEC center study was a cross-sectional study from the USA including 172 3-5-year-old children conducted in 16 ECEC centers. They found that a higher-quality outdoor play environment (e.g., shade, number of play areas, bike path quality), and more outdoor play equipment were associated with higher locomotor skills measured using Champs Motor Skills Protocol [25]. In contrast, another cross-sectional study also using Champs Motor Skills Protocol to measure 133 3-5-year-old children from 12 UK ECEC centers found that time spent in active games without use of play equipment was positively associated with higher total FMS and locomotor skills scores [21]. Active games with fixed or loose equipment were not associated with FMS in this study. Also, time spent in locomotion activities (i.e., moving while not engaged in an active play game) was negatively associated with total FMS and locomotor skills. In line with this, the third ECEC center study conducted among 229 3-5-year-old children in 22 ECEC centers also found that fixed and portable playground equipment were non-significant predictors to total gross motor scores when using Champs Motor Skills Protocol as an FMS measurement tool [26].

In a cross-sectional study from Spain, Gil-Madrona et al. [35] investigated the contribution of public playgrounds with classic features (such as slides, climbing frames, and swings) on children’s FMS - seen from a parent perspective and found that 53.7% of the 1,019 parents included in the study agreed with the positive contribution of public playgrounds to motor skills. An Italian intervention study investigated a public playground designed with specific features to promote mobility, balance and manuality. They showed a significant improvement in the experimental group of 5-year-old children (n=71) versus a control group of children (n=39) in four

out of six gross motor tasks (putting a medicine ball ($p<0.001$), one leg balance on left foot ($p<0.05$), balance on beam ($p<0.001$), and balance on platform ($p<0.001$)) after 30 minutes of structured play and 30 minutes of unstructured play once a week for a 10 weeks period [32]. In an Australian cross-sectional study, Adams et al. [29] investigated three different public playgrounds; a traditional, an adventure, and a contemporary public playground, in relation to FMS by systematically observing play in 57 children aged 5-10 at the respective playgrounds. They found that children used a wider variety of features in the contemporary and adventure playgrounds compared to the traditional playground. However, no significant associations with FMS between the three types of public playgrounds was found, possibly because a low amount of time in motor-based activities was observed. Still, the most frequently performed FMS were locomotor skills (31.3%), whereas object control skills were rarely observed (0.0-0.2%) at the three different public playgrounds [29].

Oppositely, coding of camera recordings in a Canadian cross-sectional study conducted at a rehabilitation center playground showed that six 6-7-year-old children with Down syndrome spent a great amount of time in motor-based activities (90%) in the playground setting. The primary motor activity was swinging. The tasks appeared to become more difficult as the environment became more complex (i.e., from even surface to grass and incline surface) [33]. In a cross-sectional study conducted at a rehabilitation center playground in USA among 181 3-15-year-olds both typically developed and disabled children (n=41), Miller et al. [27] coded camera recordings and found that novel use (i.e., ideation; child uses the equipment in a novel way), and motor planning (i.e., skilled, nonhabitual movements used to accomplish multistep tasks) were observed at all six playground features (sand and water table, jungle gym, Roller Slide, Mobius Climber, Cozy Dome, Omnispin Spinner). Novel use was observed most at a ‘sand and water’ table

and least at ‘Mobius Climber’ (a climbing wall). In contrast, motor planning was highest for the Mobius Climber and lowest for the ‘sand and water’ table [27].

Discussion

From the results, we realized that the 12 included publications investigated either the size, setting, and/or the features of the playground in relation to children’s FMS. In the following, we will discuss our results and highlight how future playgrounds should be designed (size, setting, and features) to support FMS development of children.

Do we need large playgrounds?

Since studies have demonstrated that children are more active in large playgrounds [36, 37], it seems obvious to conclude that more space also provides more opportunities for FMS acquisition. In the study by Grunseit et al. [28], the authors found an association between the amount of playground space available and self-reported physical activity and objectively measured fitness, but interestingly they did not find an association between playground space and FMS. Given the strong predictive association between levels of physical activity and FMS competence [5] and the positive association between playground space and both physical activity and fitness showed in the study by Grunseit et al. [28], the reason for the lack of an association between playground space and FMS is unclear. However, in the study by True et al. [26], larger playground size was significantly associated with higher total FMS score. The reason could be that the age of the children in the two studies differed. In the study by Grunseit et al. [28], the children were 5-12 years old whereas in the study by True et al. [26], the children were 3-5 years old (preschool years) which is identified as a crucial time in terms of forming and developing FMS [5]. It is therefore possible that the children in the study by Grunseit et al. [28] had past the crucial time for developing FMS lowering the influence of playground size on FMS. In fact, in another study they found that 3-

7 years-old children from rural areas with the lowest residential density had better FMS than their peers from urban areas with the highest residential density [38]. Although the focus in this study was not specifically on playgrounds, Niemiströ et al. [38] concluded that because children spend multiple hours in ECEC centers, they believe that the size of the outdoor environment near these centers (such as playgrounds) plays a notable role in children's motor development.

Do we need nature playgrounds?

Jointly, the two Norwegian studies included in this review [30, 31], indicated a positive impact of the natural environment on children's motor development. Also, a systematic review indicated some association between nature play and FMS even though this review did not focus specifically on playgrounds [39]. However, it is worth to exam[illegible] if the effect shown on green playgrounds is due to these playgrounds being placed in rural areas that might be larger and having a lower population density, as discussed above. In the study by Fjørtoft [30], the nature space used for playing by the experimental group of children was larger and herewith also lower in population density than the traditional playgrounds in the ECEC centers used by the control groups. Also, in the study by Loftesnes [31], the natural space used for building a nature playground was larger and lower in population density than the traditional playground. On the other hand, no effect of location was found in the study by Famelia et al. [34] investigating urban playgrounds in the city against rural playgrounds in farming areas in Indonesian ECEC centers. In this study, size or population density of the playgrounds were not mentioned, but it was described that limited space occurred at some settings, and they found children to be sedentary in the playgrounds around 70% of playground time, indicating that the playgrounds were relatively small. In line with this, a Norwegian study showed no differences in FMS competence of children attending nature preschools and traditional preschools [40]. This could support that playground size and density have a greater impact on FMS than nature itself. However, we know too little about how the natural

environment functions as a playground developing children's FMS to draw any conclusions on this topic.

What features do we need on the playgrounds?

In the study by Szeszulski et al. [25], the authors found both number of features and quality of features in the ECEC centers' outdoor environment to influence children's locomotor skills. On the other hand, Foweather et al. [21] found that time spent in active games without equipment was positively associated with higher locomotor skills score and total FMS. This finding suggests that spending more time on active games such as dancing, chasing games, and rough and tumble play without use of playground features may be important for FMS development. Also, previous research has demonstrated that preschool children in the highest locomotor skill tertile generally engaged in more dancing than children in the lowest tertile [41]. In the study by Foweather et al. [21], however, children spent a relatively large proportion of time (41%) engaged in active games with equipment, but this type of play was not associated with FMS, possibly because the children were frequently observed being sedentary on the equipment. It is possible that these pieces of equipment supported other FMS capacities, such as climbing or stability skills, not assessed in the study by Foweather et al. [21]. Nevertheless, this finding is similar to Adams et al. [29] reporting that the children used a wider variety of equipment in the contemporary and adventure playgrounds than the traditional playground, but they did not find a statistically significant association between the FMS observed at the three playgrounds varying in features. Also, these authors suggest that it is possible that the general low FMS mastery among children could be influenced by the lack of FMS required to play in playgrounds [29].

From the studies, however, various features seem to encourage varying motor competences making it complex to answer exactly what features are needed in the playground to

improve children's FMS development. In the study by Adams et al. [29], locomotor skills such as walking and running were observed most frequently in the contemporary playground where the features were spread over a large area requiring children to use locomotor skills to move around. Conversely, locomotor skills were observed less frequently at the adventure playground where the features were linked off a large walkway and children needed different FMS to move to and from different features such as balancing. Still, climbing nets were the most used play feature at the adventure playground also stimulating locomotor skills [29]. Climbing and hanging features are also important to develop upper-body strength [42]. Importantly, Adams et al. [29] and True et al. [26] found no association between playground play and object control skills. According to True et al. [26], features to improve object control skills seems not to be provided very often in playgrounds for preschool children. Also, a study found object control skills to develop at a slow rate before the age of 9-10 [43]. Portable features such as balls might influence object control skills. Portable features, however, was not studied in the current review. As Tortella et al. [32] showed, specifically targeted playground equipment may be necessary to encourage FMS development. The authors conclude, however, that specific training using specific playground features, only affects the development of that task trained and not necessarily other tasks related to the same FMS competence [32]. This conclusion is supported by Revie and Larkin [16] investigating the effects of eight sessions of intensive teaching of FMS in children with poor coordination.

A sensory-rich playground provided with varied features, enticing colors, and multitextured materials also seem to be valuable for the development of children with disabilities [27]. However, according to Virji-Babul et al. [33] children with disabilities seem to have more difficulties in extracting and processing relevant information from the physical environment than children traditional developed, leading to decreased engagement in free play at the playground.

Thus, it also seems important that playground features are easy to interpret and can be used at different developmental stages.

Strengths and limitations

We followed a robust review protocol, thus the risk of bias in our review methodology is low [23]. Further, a strength is that the search procedure was developed by a research group of experts in the research field of playground usage in collaboration with a librarian with huge expertise in search strategies. To capture as much relevant research, four different databases were searched. However, given the large number of publications retrieved, we questioned if we should have created a third block containing health outcomes to narrow-down our search[illegible] Also, no quality assessment of included publications was performed. Since only 12 publications were included in the present scoping review, we wanted to cover all knowledge on the subject regardless of the design and quality of the study. A challenge was that the publications used many different child-monitoring instruments to measure FMS, possibly because there is little agreement on what FMS measurement should be used [44].

Conclusion and future directions

The aim of the current scoping review was to create an overview of all research that is relevant when studying the influence of unstructured playground play on children's FMS. Twelve studies investigated unstructured playground play and children's FMS. From the current scoping review, it seems important to design playgrounds with various features targeting balance, climbing, throwing, and catching to provide opportunities for children to enhance each FMS (i.e., stability, locomotor skills, and object control skills). Also, spreading features over a large area seems to both ensure ample space per child and to stimulate children to use locomotor skills by moving to and from features and by playing active games without equipment. Possibly, also natural play settings

develop children’s FMS. Our results, however, should be read with caution. Overall, based on only 12 studies reviewed, we still know too little about the association between unstructured playground play and FMS, and more effort should be dedicated to future studies in this field. In particular, we need more experimental studies using objective and standardized FMS tests since only two of the 12 studies had this high-quality design. Therefore, it is needed also to discuss the quality of the used FMS tests in future research.

Acknowledgement

We thank academic officer Danielle Nørager Johansen from University of Southern Denmark who helped organizing and coordinating the search and extraction process. Also, thanks to librarian Lasse Østergaard from University of Southern Denmark who helped with the search strategi.

References

1. Hulteen RM, Morgan PJ, Barnett LM, Stodden DF, Lubans DR. Development of Foundational Movement Skills: A Conceptual Model for Physical Activity Across the Lifespan. Sports Medicine. 2018; 48(7): 1533-1540.
2. Xin F, Chen ST, Clark C, Hong JT, Liu Y, Cai YJ. Relationship between Fundamental Movement Skills and Physical Activity in Preschool-aged Children: A Systematic Review. International Journal of Environmental Research and Public Health. 2020; 17(10).
3. Lima RA, Pfeiffer K, Larsen LR, Bugge A, Moller NC, Anderson LB, et al. Physical Activity and Motor Competence Present a Positive Reciprocal Longitudinal Relationship Across Childhood and Early Adolescence. Journal of Physical Activity and Health. 2017; 14(6): 440-447.
4. Robinson LE, Stodden DF, Barnett LM, Lopes VP, Logan SW, Rodrigues LP, et al. Motor Competence and its Effect on Positive Developmental Trajectories of Health. Sports Medicine. 2015; 45(9): 1273-1284.
5. Hardy LL, Reinten-Reynolds T, Espinel P, Zask A, Okely AD. Prevalence and Correlates of Low Fundamental Movement Skill Competency in Children. Pediatrics. 2012; 130(2): E390-E398.
6. Bolger LE, Bolger LA, O’Neill C, Coughlan E, O’Brien W, Lacey S, et al. Global levels of fundamental motor skills in children: A systematic review. Journal of Sports Science. 2021; 39(7): 717-753.
7. Ericsson I, Karlsson MK. Motor skills and school performance in children with daily physical education in school-a 9-year intervention study. Scandinavian Journal of Medicine and Science in Sports. 2014; 24(2): 273-8.

8. van der Fels IM, Te Wierike SC, Hartman E, Elferink-Gemser MT, Smith J, Visscher C. The relationship between motor skills and cognitive skills in 4-16 year old typically developing children: A systematic review. Journal of Science and Medicine in Sport. 2015; 18(6): 697-703.
9. Herrmann C, Bretz K, Kühnis J, Seelig H, Keller R, Ferrari I. Connection between Social Relationships and Basic Motor Competencies in Early Childhood. Children-Basel. 2021; 8(1).
10. Vedul-Kjelsås V, Sigmundsson H, Stensdotter AK, Haga M. The relationship between motor competence, physical fitness and self-perception in children. Child: Care, Health and Development. 2012; 38(3): 394-402.
11. Lai SK, Costigan SA, Morgan PJ, Lubans DR, Stodden DF, Salmon J, et al. Do School-Based Interventions Focusing on Physical Activity, Fitness, or Fundamental Movement Skill Competency Produce a Sustained Impact in These Outcomes in Children and Adolescents? A Systematic Review of Follow-Up Studies. Sports Medicine. 2014; 44(1): 67-79.
12. Morgan PJ, Barnett LM, Cliff DP, Okely AD, Scott HA, Cohen KE, et al. Fundamental movement skill interventions in youth: a systematic review and meta-analysis. Pediatrics. 2013; 132(5): e1361-83.
13. Jiménez-Díaz J, Chaves-Castro K, Salazar W. Effects of Different Movement Programs on Motor Competence: A Systematic Review With Meta-Analysis. Journal of Physical Activity and Health. 2019; 16(8): 657-666.
14. Ma J, Hogan MJ, Eyre EL, Lander N, Barnett LM, Duncan MJ. Enhancing the implementation and sustainability of fundamental movement skill interventions in the UK and Ireland: lessons from collective intelligence engagement with stakeholders. International Journal of Behavioral Nutrition and Physical Activity. 2021; 18(1).
15. Logan SW, Robinson LE, Wilson AE, Lucas WA. Getting the fundamentals of movement: a meta-analysis of the effectiveness of motor skill interventions in children. Child: Care, Health and Development. 2012; 38(3): 305-15.
16. Revie G, Larkin D. Task specific intervention with children reduces movement problems. Adapted Physical Activity Quarterly. 1993, 10: 29-41.
17. Berg, S. Children's Activity Levels in Different Playground Environments: An Observational Study in Four Canadian Preschools. Early Childhood Education Journal. 2015; 43(4): 281-287.
18. Bento G, Dias G. The importance of outdoor play for young children's healthy development. Porto Biomedical Journal. 2017; 2(5): 157-160.
19. World Health Organization. Guidelines on physical activity, sedentary behaviour and sleep for children under 5 years of age. 2019; License: CC BY-NC-SA 3.0 IGO: [Available from: https://apps.who.int/iris/handle/10665/311664.
20. Johnstone A, Hughes AR, Martin A, Reilly JJ. Utilising active play interventions to promote physical activity and improve fundamental movement skills in children: a systematic review and meta-analysis. BMC Public Health. 2018; 18(1): 789.
21. Foweather L, Crotti M, Foulkes JD, O'Dwyer MV, Utesch T, Knowles ZR, et al. Foundational Movement Skills and Play Behaviors during Recess among Preschool Children: A Compositional Analysis. Children-Basel. 2021; 8(7).
22. Parrish AM, Yeatman H, Iverson D, Russell K. Using interviews and peer pairs to better understand how school environments affect young children's playground physical activity levels: a qualitative study. Health Education Research. 2012; 27(2): 269-80.

23. Peters MDJ, Godfrey C, McInerney P, Munn Z, Tricco AC, Khalil H. Chapter 11: Scoping reviews, in JBI Manual for Evidence Synthesis, E. Aromataris and Z. Munn, Editors. JBI; 2020
24. PRISMA for Scoping Reviews. 2018; Available from: www.prisma-statement.org/Extensions/ScopingReviews.
25. Szeszulski J, Lorenzo E, Todd M, O'Connor TM, Hill J, Shaibi GQ, et al. Early Care and Education Center Environmental Factors Associated with Product- and Process-Based Locomotor Outcomes in Preschool-Age Children. International Journal of Environmental Research and Public Health. 2022. 19(4).
26. True L, Pfeiffer KA, Dowda M, Williams HG, Brown WH, O'Neill JR, et al. Motor competence and characteristics within the preschool environment. Journal of Science and Medicine in Sport. 2017; 20(8): 751-755.
27. Miller LJ, Schoen SA, Camarata SM, McConkey J, Kanics IM, Valdez A, et al. Play in natural environments: A pilot study quantifying the behavior of children on playground equipment. Journal of Occupational Therapy, Schools, & Early Intervention. 2017; 10(3): 213-231.
28. Grunseit AC, O'Hara BJ, Drayton B, Learnihan V, Hardy LL, Clark E, et al., Ecological study of playground space and physical activity among primary school children. BMJ Open. 2020; 10(6).
29. Adams J, Veitch J, Barnett L. Physical Activity and Fundamental Motor Skill Performance of 5-10 Year Old Children in Three Different Playgrounds. International Journal of Environmental Research and Public Health. 2018; 15(9).
30. Fjørtoft I. The Natural Environment as a Playground for Children: The Impact of Outdoor Play Activities in Pre-Primary School Children. Early Childhood Education Journal. 2001; 29: 111-117.
31. Loftesnes JM, In the nature playground - initiation and evaluation of the project at 9 kindergartens. Pitesti. 2021; Feb: 542-548.
32. Tortella P, Haga M, Loras H, Sigmundsson H, Fumagalli G. Motor Skill Development in Italian Pre-School Children Induced by Structured Activities in a Specific Playground. PLoS One. 2016; 11(7): e0160244.
33. Virji-Babul N, Hovorka R, Jobling A. Playground dynamics: Perceptual-motor behaviour and peer interactions of young children with Down syndrome. Journal on Developmental Disabilities. 2006; 12(1): 29-44.
34. Famelia R, Tsuda E, Bakhtiar S, Goodway J. Relationships among perceived and actual motor skill competence and physical activity in Indonesian preschoolers. Journal of Motor Learning and Development. 2018; 6: 1-12.
35. Gil-Madrona P, Martínez-López M, Prieto-Ayuso A, Saraiva L, Vecina-Cifuentes J, Vicente-Ballesteros T, et al. Contribution of Public Playgrounds to Motor, Social, and Creative Development and Obesity Reduction in Children. Sustainability. 2019; 11(14).
36. Worobey J, Worobey HS, Adler AL. Diet, activity and BMI in preschool-aged children: Differences across settings. Ecology of Food and Nutrition. 2005; 44(6): 455-466.
37. Escalante Y, Backx K, Saavedra JM, García-Hermoso A, Dominguez AM. Play area and physical activity in recess in primary schools. Kinesiology: international journal of fundamental and applied kinesiology. 2012; 44: 123-9.
38. Niemistö D, Finni T, Haapala EA, Cantell M, Korhonen E, Sääkslahti A. Environmental Correlates of Motor Competence in Children-The Skilled Kids Study. International Journal of Environmental Research in Public Health. 2019; 16(11).

39. Dankiw KA, Tsiros MD, Baldock KL, Kumar S. The impacts of unstructured nature play on health in early childhood development: A systematic review. PLoS One. 2020; 15(2): e0229006.
40. Lysklett OB, Berg A, Moe B. Motor competence and physical fitness among children attending nature preschools and traditional preschools. International Journal of Play. 2019; 8(1): 53-64.
41. O'Neill JR, Williams HG, Pfeiffer KA, Dowda M, McIver KL, Brown WH, et al. Young children's motor skill performance: relationships with activity types and parent perception of athletic competence. Journal of Science in Medicine and Sport. 2014; 17(6): 607-10.
42. Suttervy J, Thornton C. It doesn’t just happen! Essential contributions from playgrounds. Young Children. 2005; 60: 26-33.
43. Butterfield SA, Angell RM, Mason CA. Age and sex differences in object control skills by children ages 5 to 14. Perceptual and Motor Skills. 2012; 114(1): 261-74.
44. Haywood KM, Getchell N. Life span motor development. 4th ed. Human Kinetics; 2005

Table 1. Included publications studying the influence of unstructured playground play on children's fundamental movement skills.

**Author, year, and country**	**Study aim**	**Study design**	**Population**	**Setting**	**Health outcome(s)**	**FMS measurement**	**FMS Result(s)**
Adams et al (2018), Australia [29]	Whether playground design facilitated different levels of PA and FMS	Cross-sectional	57 children, 5-10 yrs.	Neighborhood (3 playgrounds in 1 city; traditional, adventure, contemporary).	FMS + PA	SOFIT (modified)	There were no significant associations with FMS between the three playgrounds.<br><br>The most frequent performed FMS were locomotor skills (31.3%), specifically walking (18.3%) and running (11.3%). Body management skills (15.2%) and climbing (12.3%) was also observed at all three playgrounds, whereas object control skills such as catching and throwing were rarely observed (0.0-0.2%)<br><br>Children performed few FMS but used a wider variety of equipment in the

							contemporary and adventure playgrounds.
Famelia et al (2018), Indonesia [34]	Relationships among FMS, playground PA, and gender	Cross-sectional	66 children, 3-6 yrs.	4 childcare centers (2 urban + 2 rural)	FMS + PA	PMSC + the Test of Gross Motor Development-3	No main effect of location for locomotor skills and perceived movement skill competence.
Fjørtoft (2001), Norway [30]	How playing in the natural environment might stimulate FMS	Intervention (play in forest versus traditional playground 1-2 hours/day for 9 months)	75 children, 5-7 yrs. (46 in the experimental group)	3 childcare centers (1 experimental + 2 control)	FMS	EUROFIT + Beam walking and Indian skip	At the posttest 9 months after the pretest, significant differences were found in eight out of nine FMS test items in the experimental group (flamingo balance ($p<0.001$), plate tapping ($p<0.001$), standing broad jump ($p<0.001$), bent arm hang ($p<0.001$), Indian skip ($p<0.001$), sit-ups ($p<0.01$), beam walking ($p<0.01$), and shuttle run ($p<0.01$)) whereas the control group experienced a significant difference in three test items (standing

							broad jump (p<0.01), bent arm hang (p<0.001), and Indian skip (p<0.001).<br><br>At the pretest the experimental group scored lower than the control group, but scored better in all test items at the posttest
Foweather et al (2021), England [21]	The association between play behavior and FMS during recess at preschool	Cross-sectional	133 children, 3-5 yrs.	12 childcare centers	FMS	Video-assessment using CMSP	Relative to time spent in other types of play behaviors, time spent in play without equipment was positively associated with total FMS and locomotor skills, while time spent in locomotion activities (moving while not engaged in an active play game) was negatively associated with total FMS and locomotor skills
Gil-Madrona et al	The contribution of public playgrounds to	Cross-sectional	1019 adults	Neighborhood (41 parks in 1 city)	FMS, weight status, and social +	Survey	53.7% parents agreed with the positive

(2019), Spain [35]	obesity reduction, motor, social, and creative development				creative skills		contribution of public playgrounds to motor skills (38% quite agree and 15.7% totally agree).
Grunseit et al (2020), Australia [28]	Relationship between school playground size and PA, fitness, and FMS	Cross-sectional	5238 children, 5-12 yrs.	43 primary schools	FMS, PA, Physical fitness	Scoring of 7 FMS skills	No association between playground space and motor skills.
Loftesnes (2021), Norway [31]	Evaluating a new-built nature playground for children aged 2-6 years	Cross-sectional post-intervention study (Build a nature playground and use it for min 2 days a week for an entire year)	30 adults (18 staff + 12 parents)	9 childcare centers	FMS, social skills	Survey + interview	Parents found their child being more able to cope with motor skills.
Miller et al (2017), USA [27]	Quantify equipment/areas impacted for children with sensory challenges	Cross-sectional	181 children, 3-15 yrs. (41 disabled)	child rehabilitation center (1 playground)	FMS, social skills	Coding of camera recordings	The behavior most often observed across all pieces of equipment was novel use, ranging from 41.82-97.66% of the time. Least = Mobius, most = sand and water. Motor planning was highest for the Mobius Climber (58.18%) and lowest for sand and water.

Szeszulski et al (2022), USA [25]	Association between the characteristics of the childcare center environment and FMS	Cross-sectional	172 children, 3-5 yrs.	16 childcare centers	FMS, PA	PACER (product-based locomotor skills) CMSP (process-based locomotor skills)	Better outdoor play environment quality score and more outdoor equipment were positively associated with higher CMSP scores.
Tortella et al (2016), Italy [32]	Effects of structured and unstructured activities played at the playground on FMS	Intervention (10 weeks – 1 hour half structured and half unstructured play at specific playground)	110 children, 5 yrs. (71 in the experimental group)	Neighborhood (1 playground)	FMS	Scoring of 9 FMS skills (3 for fine and 6 for gross motor skills)	The experimental group improved significantly in 4 out of 6 gross motor tasks (putting a medicine ball ($p<0.001$), one leg balance on left foot ($p<0.05$), balance on beam ($p<0.001$), and balance on platform ($p<0.001$)) and in none of the fine motor tasks.
True et al (2017), USA [26]	The contribution of various preschool environmental characteristics to children's FMS	Cross-sectional	229 children, 3-5 yrs.	22 childcare centers (4 head start, 7 faith-based, 11 commercial)	FMS, PA	CMSP	Playground size is a significant predictor of total motor score (effect size 0.33) when adjusting the analyses for other significant predictors, e.g., age, classroom size, teacher education and electronic

							media use but not locomotor score and object control score, individually.<br><br>Time spent in outdoor open spaces, fixed and portable playground equipment were non-significant predictors to total gross motor scores.
Virji-Babul et al (2006), Canada [33]	Analyzing the level of motor engagement within the playground	Cross-sectional	6 children with DS, 6-7 yrs.	1 Child rehabilitation center	FMS + social skills	Coding of camera recordings	Children spent a great amount of time in motor-based activities (90%) in a playground setting.<br><br>The primary motor activity was swinging. The tasks appeared to become more difficult as the environment became more complex (even surface versus grass and incline surface).

CMSP=the Champs Motor Skill Protocol; DS=Downs syndrome; EUROFIT= European Test of Physical Fitness, the Motor Fitness Test; FMS=fundamental movement skills; PA=physical activity; PACER=the Progressive Aerobic Cardiovascular Endurance Run; PMSC= Pictorial Scale of Perceived Movement Skill Competence; yrs.=years; SOFIT=System for Observing Fitness Instruction Time

Figure 2 Flowchart for selected publications

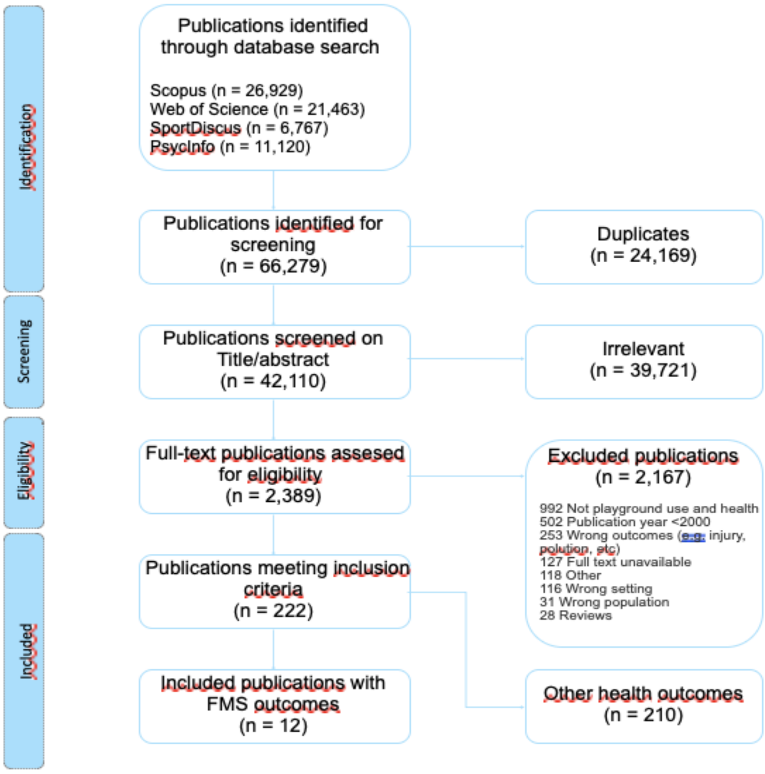

PRISMA checklist

Click here to access/download
Supporting Information
PRISMA-Checklist.docx
