# Supplementary material for: The role of playgrounds in the development of children’s fundamental movement skills: A scoping review
Source: PLoS One. 2023 Dec 13;18(12):e0294296. doi: 10.1371/journal.pone.0294296 (PMC10718446; doi:10.1371/journal.pone.0294296)
Supplement: Attachment — Submitted filename: Response to reviewer.docx [file pone.0294296.s002.docx]

**Response to reviewers**

| No. | Editor comment | Author respons |
| --- | --- | --- |
| 1 | Please ensure that your manuscript meets PLOS ONE's style requirements, including those for file naming. | We have corrected file names |
| 2 | We note that the grant information you provided in the ‘Funding Information’ and ‘Financial Disclosure’ sections do not match. When you resubmit, please ensure that you provide the correct grant numbers for the awards you received for your study in the ‘Funding Information’ section. | We do not have any grant numbers from the funders who did not specifically funding this article/project but funds the institute that did the paper work. See our comments below. |
| 3 | 3. Please address the following queries:  a)        Please clarify the sources of funding (financial or material support) for your study. List the grants or organizations that supported your study, including funding received from your institution.  b)        State what role the funders took in the study. If the funders had no role in your study, please state: “The funders had no role in study design, data collection and analysis, decision to publish, or preparation of the manuscript.”  c)        If any authors received a salary from any of your funders, please state which authors and which funders.  d)        If you did not receive any funding for this study, please state: “The authors received no specific funding for this work.”   Please include your amended statements within your cover letter; we will change the online submission form on your behalf. | Our study is funded by Kompan A/S who has funded the World Playground Research Institute at University of Southern Denmark. The authors Charlotte Skau Pawlowski, Mette Toftager, and Jasper Schipperijn are employed part time in the World Playground Research Institute and receive 50% of their salary from Kompan A/S and 50% of their salary from University of Southern Denmark. Cathrine Damsbo Madsen is employed fulltime in the World Playground Research Institute and receive 100% of her salary from Kompan A/S. The funders had no role in study design, data collection and analysis, decision to publish, or preparation of the manuscript. |
| 4 | Please complete your Competing Interests on the online submission form to state any Competing Interests. If you have no competing interests, please state "The authors have declared that no competing interests exist."  This information should be included in your cover letter; we will change the online submission form on your behalf. | "The authors have declared that no competing interests exist." |
| 5 | In your Data Availability statement, you have not specified where the minimal data set underlying the results described in your manuscript can be found. PLOS defines a study's minimal data set as the underlying data used to reach the conclusions drawn in the manuscript and any additional data required to replicate the reported study findings in their entirety. All PLOS journals require that the minimal data set be made fully available.  Upon re-submitting your revised manuscript, please upload your study’s minimal underlying data set as either Supporting Information files or to a stable, public repository and include the relevant URLs, DOIs, or accession numbers within your revised cover letter.  Important: If there are ethical or legal restrictions to sharing your data publicly, please explain these restrictions in detail. Please see our guidelines for more information on what we consider unacceptable restrictions to publicly sharing data.  We will update your Data Availability statement to reflect the information you provide in your cover letter. | This paper is a scoping review. Thus, our analysis is a meta-analysis relying on published third-party data. All data used for the meta-analysis is provided in the manuscript, Table 1. In Table 1, information on each published paper, used as data in the manuscript, can be found. |
| 6 | Please include captions for your Supporting Information files at the end of your manuscript, and update any in-text citations to match accordingly. | We have aligned table number and caption for table 2 in-text and at the end of our manuscript. |
| 7 | Please review your reference list to ensure that it is complete and correct. If you have cited papers that have been retracted, please include the rationale for doing so in the manuscript text, or remove these references and replace them with relevant current references. Any changes to the reference list should be mentioned in the rebuttal letter that accompanies your revised manuscript. If you need to cite a retracted article, indicate the article’s retracted status in the References list and also include a citation and full reference for the retraction notice | We have not cited papers that have been retracted. |
