# Supplementary material for: The role of playgrounds in the development of children’s fundamental movement skills: A scoping review
Source: PLoS One. 2023 Dec 13;18(12):e0294296. doi: 10.1371/journal.pone.0294296 (PMC10718446; doi:10.1371/journal.pone.0294296)
Supplement: Attachment — Submitted filename: Response to Reviewers.docx [file pone.0294296.s004.docx]

**Response to Reviewers**

| No. | Reviewer comments | Author responses |
| --- | --- | --- |
| 1 | Line 37 - This is a firm statement with only one reference to support the statement. Could this be rephrased to state something like: FMS have previously been shown to be low in preschool and school aged children from high-income countries. | Good idea. The sentence has been reworded as suggested, lines 36-37. |
| 2 | Line 48 - For clarity, please replace the word 'this' with exactly what you are referring to. | We have reworded the sentence |
| 3 | Line 50 - The word 'also' is over used in this manuscript. Please consider editing the manuscript to reduce the number of times it is required. | We have decreased the use of the word “also” |
| 4 | Line 56 - Please insert 'systematic' in front of the word 'review'. | Done |
| 5 | Line 58 - Please insert full-stop after the word 'heterogeneity'. | Done |
| 6 | Line 61 - Please replace the word 'article' with 'scoping review'. | Done |
| 7 | Line 62 - It appears in the way you have presented your results that you had some specific aims (e.g. impact of playground size, location etc). If this is correct, could you please more accurately state your aims? | We hope we have made our study more clear by connecting the following key themes investigated to the aim of the study: playground size, setting and features, lines 63-64. |
| 8 |  | We have used the PRISMA extension for scoping reviews (PRISMA-ScR) and added the reference line 69. |
| 9 | Line 68 - Please confirm if you have utilised the PRISMA extension for scoping reviews (PRISMA-ScR).  Tricco, AC, Lillie, E, Zarin, W, O'Brien, KK, Colquhoun, H, Levac, D, Moher, D, Peters, MD, Horsley, T, Weeks, L, Hempel, S et al. PRISMA extension for scoping reviews (PRISMA-ScR): checklist and explanation. Ann Intern Med. 2018,169(7):467-473. doi:10.7326/M18-0850. | Sure, we have made a second search see our new search strategy described in lines 82-86. New information about the second search is also described in Table 1 + figure 1. Two more papers from this second search are included in the review. |
| 10 | Line 109 - Currently you jump back and forth between inclusions and exclusions. Could you please reorder this content to have the inclusions and exclusions grouped together for improved clarity? | Good idea – We have reordered the content to have the inclusions and exclusions grouped together and hope this has improved clarity, lines 90-104. |
| 11 | Line 154 - Please check Table numbers. At the end of the manuscript you list Table 1 (not 2) as the data extraction table. | Thank you for being aware of that mistake. We have corrected the Table at the end of the manuscript to Table 2. |
| 12 | Line 160 - Can you provide some rationale for your dissecting the publications by decades? Are the decades meaningful by decades (new rules / policy etc). Why is this information important for the reader to know about? How does it relate to your aims? You may need to put some information in your methods about this step of data synthesis. | No, you are right, we did not have a rationale for our dissecting the publications by decades. Therefore, we have now deleted this section. |
| 13 | Line 165 - Could you please rewrite these characteristics summaries to refer to studies. I.e., the publications were not undertaken in early childhood education centers but rather the studies were. | Good point – we have reworded from publication to study throughout the manuscript where mentioning study instead of publication made more sense. |
| 14 | Line 168 - Here you state 2 studies included adults. Did they also include children 0-17? (As in your inclusion criteria you state you were including publications examining children aged 0-17 years). If this study did also included children 0-17 years, then in your methods, you will need to be clear that you will accept studies / publications where 0-17 year olds are included in addition to adults. However, if these two studies are just about adults, then according to your inclusion criteria you should exclude them. It is unclear to me if these two studies with adults in ref 35 and 31 or not? | We realize that we have not been clear on which participants that were included in the scoping review. Thanks for being aware of this. We have tried to clarify this by adding following sentence to the selection criteria section: “Studies were also included if the participants were parents or professionals around children” (lines 95-96). |
| 15 | Line 172 - Were the children actually disabled? Or did they have a condition / impairments that led to atypical development? Please consider the use of the word 'disabled' and if it is the correct use of this term (I am not sure, it may or may not be, but the use of this language should carefully considered in line with ICF language expectations). | Thanks for this comment – We have reworded disabled children to children with special needs that have led to atypical development. The publication referred (Miller et al 2017) also use this term. |
| 16 | Line 192 - I am not sure what is meant by this sentence. Loftesnes is not an English Lanague word (to my knowledge). Could you please help the English language readers to better understand the meaning of this sentence? ADDITIONAL STATEMENT: I think from reading the discussion this is actually an author (Please add the reference citation for the author). | Sorry for making this confusion. I have added the reference, line 193. |
| 17 | Line 217 - Please correct to post-test | Corrected |
| 18 | Line 228 - Please see statement above about the use of the word 'disabled' children. If the children were in fact 'disabled' please use person first language in line with expectations of ICF. E.g. Children with diagnosed conditions resulting in disability.  Not all children with impairments will be 'disabled'. | Thanks for this comment – We have reworded disabled children to children with special needs that have led to atypical development. |
| 19 | Line 234 - Please change 'spend' to past tense 'spent' | Changed |
| 20 | Line 235 - Are you able to provide more information about the 'play equipment' here...so that the reader can better interpret the findings? | We have added more information on the different play equipment terms (lines 221-223). We have added the examples used in the study by Foweather et al 2021 to define the different play equipment terms. |
| 21 | Line 251 - Replace 'at' with 'a' | Changed |
| 22 | line 254 - insert years after 5-10. | We have added the word years after 5-10 |
| 23 | Line 267 - As above for statement - change to person first language. | Thanks for this comment – We have reworded disabled children to children with special needs atypically development. |
| 24 | Lines 275 - 276: If this was not connected with your original aims, then I would encourage you to write something about these key themes prior to presenting them, in your results section. | We hope we have made our study more clear by connecting the following key themes investigated to the aim of the study: playground size, setting and features. See also our response to comment 7. Thus, the introduction to the discussion has also been reworded not mentioning that these key themes were found in the results. |
| 25 | Line 283 - Replace 'objective' with 'objectively' | Changed |
| 26 | Line 304 - Please replace 'worth to examine' with 'worth examining' | Changed |
| 27 | Line 364 - Please replace 'traditional' with 'traditionally' | Changed to typically for alignment |
| 28 | Line 368 - Please provide some detail about the robust features of your methods. | We have provided some details about the robust features of our methods and hopefully made it more clear what we meant about a robust protocol in lines 359-360. |
| 29 | Line 373 - This is a nice insight to share. | Thank you |
| 30 | Line 531 - For Table 1 - Please consider reformatting so that it can be included in the manuscript. Currently the final right hand side column has most relevant content. You could merge the FMS headings and in the column put sub headings:  FMS Measurement:  FMS Result:  Also the same for  Study Aim:  Study Design:  and  Population:  Setting:  This way your table can more easily be included with the manuscript without a lot of empty space. | Very good idea – thank you for that advice. I have changed Table 1 as suggested. |
